# Another Remarkable Obstetrical Case

**Published:** 1859-07

**Authors:** 


### *Another Remarkable Obstetrical Case.*

Prof. Austin Flint, Jr., Editor, &c., &c.:

*Dear Sir,*—I am much gratified in being the medium of transmission, for the pages of your valuable journal, of another amusing and instructive case of false diagnosis in midwifery. It will doubtless bring to the mind of every one engaged extensively in the practice of obstetrics, many similar blunders, which have fallen under his own observation. It may be added, that these errors are, to a certain extent, the legitimate result of sending young men out to practice midwifery, without the least opportunity for clinical instruction. Simply witnessing, at the bedside, under competent instruction, a single labor, would do more towards preventing such awkward and dangerous mistakes, than can ever be accomplished by any amount of theoretical teaching alone. The neophyte must observe the process of parturition in the living subject, and make himself familiar with the relative changes between the presenting part of the child and the organs of the mother, as they occur.

Such blunders in the practice of regular members of the profession, who have been sent forth from the schools, to acquire, unaided, upon private patients, and often at the hazard of life, that knowledge which should have been supplied them by their teachers, before conferring their degrees, affords conclusive proof of the necessity of clinical teaching in this department, or, in other words, of the necessity of demonstrative midwifery.

But, without farther comment, I send you the report of the following case, which is furnished me by one of the oldest and most respectable practitioners in the State.

Yours truly,

JAMES P. WHITE.

13TH APRIL, 1859.

JAMES P. WHITE, M. D.:

*My Dear Doctor*,—Your "Remarkable Obstetrical Case," in the last number of the Buffalo Medical Journal, reminds me, in several particulars, of a somewhat similar case, which came under my own observation.

In the month of March, 1848, Mr. M——, of an adjoining town, came for me, with a horse and buggy, the second time within a few hours (the first time I could not go), to meet three gentlemen in consultation, in a case of midwifery, and to take "my instruments" with me. He was anxious that I should not delay, as he was very fearful it might be too late to render his wife any assistance. Placing the "green bag" under the seat, I immediately accompanied him; and, on the way, received from him, in substance, the following relation: Mrs. M—— was his second wife; they had been married about a year; this was her first child; her age was about thirty-five; she had already been in labor nearly five days; Dr. No. 1, the first called, had been in attendance a day and a half. He said there was no opening to the womb discoverable; and he was afraid, if the pains kept up, womb and all would be forced into the world. Indeed, he said, it was as much as he could do, at times, to prevent it. Mr. M. said "he believed it had come out once or twice, and the Dr. had pushed it back again,—at least, so the women said." He wanted counsel, and they then called in Dr. No. 2. He had been in attendance nearly two days, and agreed with Dr. No. 1, but thought some operation was necessary; and requesting further advice; then we called in Dr. No. 3, who, with the other two, had been in attendance several hours; and, no favorable change taking place, they all agreed that something must be done, and had concluded to cut open the womb. Mr. M. said he was unwilling to have it done until I had passed my judgment upon the case, "for, to tell you the truth, Dr.," said he, "I don't believe any of them begin to know what the matter is."

When I reached the house, I found only one of the gentlemen there—Dr. No. 1. He was fast asleep in a rocking-chair—the exhausting labors of three or four nights' attendance predisposing to sleep; he seemed to be in close communion with "nature's sweet restorer;" and who could blame him? Several ladies, as usual, were in attendance, who, in the main, confirmed the above facts. Their countenances expressed varied shades of anxiety and distress. I was requested by them to make an examination at once; but of course I declined until the arrival of the other gentlemen. The "pains," I judged, were frequent, urgent, and protracted. Soon Dr. No. 1 began to rub open his eyes, and stretch out awake. Of course, he was glad to see me. From him I received about the same statement as from Mr. M.—only a little more technical, of course. He said it was a case of *occlusion of the os tincæ;* and they were fearful of a rupture of the uterus, unless they made an immediate incision, and extracted the child. Drs. No. 2 and 3, he said, had been in attendance with him, and they all agreed in theory and practice; that they had now gone to Dr. No. 2's office, to procure the necessary instruments, and would soon be back. He asked me to take off my coat, and make an examination. At first, I hesitated; for I wanted to see the united council together; but the Dr. insisted, and the ladies entreated, and I consented. And what think you was my surprise, when I found a hard, unyielding substance protruding just within the vulva—pressing hard upon the perineum,—with perfect dilatation, and apparently labor just about terminating! I whispered to the Dr. the result of my examination, and requested him to examine again, to see if a change had not recently occurred. He did so, and said there was no change at all; that I had mistaken the womb itself for the head of the child. He expressed his opinion so confidently, and so audibly, that the ladies present, including the patient, understood not only that we differed in opinion, but in what that difference consisted. Privacy, of course, now became impossible. I asked him if he did not feel the hair on the scalp. He laughed at me for such a suggestion. I told him the eye would test it very quick, if the lady would consent. She readily yielded to the request, when lo! there it was, sure enough,—scalp, hair, and all. Just at this juncture, but not until I had encouraged the patient, by assuring her that all would soon be well, without any operation, Drs. No. 2 and 3 entered the room. The usual salutations were few and short. Dr. No. 1 announced the discovery. Never were men more completely "dumb-founded." At first, they would not believe it; not until they saw the hair itself on the scalp. But now there was no time for parley or delay. Nature would no longer be trifled with. She had taken the case into her own hands; and, in less time than it has taken me to tell you the story, a large, well-formed, still-born infant was ushered into the world. It would have taken more than the pencil of a Hogarth, or the pen of a Swift, to have given an adequate representation of the scene ensuing. I shall not attempt it. Your imagination, if ever so fertile, can't exceed it.

If our young friend would like to give this case juxtaposition with its "remarkable" fellow, in his very readable Journal, I have no objection. I agree with him in, at times, feeling "ashamed of our race, because they will suffer such things; and of our profession, because we can't prevent them." It has long since passed into a proverb, that "that which cannot be cured, must be endured." If the occasional publication of such cases can't *cure* the evil, I think it is decidedly prophylactic in its tendency; and their perusal affords an occasional relief to the anxiety and severity of our daily professional toil.

A LICENTIATE OF 1813.

